# Computational Design of Photosensitive Polymer Templates To Drive Molecular Nanofabrication

**DOI:** 10.1021/acsnano.3c10575

**Published:** 2024-03-28

**Authors:** Mithun Manikandan, Paolo Nicolini, Prokop Hapala

**Affiliations:** Institute of Physics (FZU), Czech Academy of Sciences, Na Slovance 2, 182 00 Prague, Czech Republic

**Keywords:** nanofabrication, computational screening, DNA analogue, hydrogen bonded system, self-assembly, *ab initio* calculations, molecular electronics

## Abstract

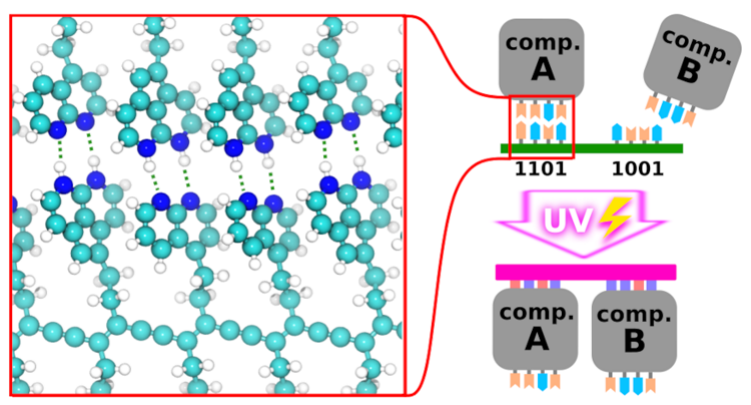

Molecular electronics promises the ultimate level of miniaturization of computers and other machines as organic molecules are the smallest known physical objects with nontrivial structure and function. But despite the plethora of molecular switches, memories, and motors developed during the almost 50-years long history of molecular electronics, mass production of molecular computers is still an elusive goal. This is mostly due to the lack of scalable nanofabrication methods capable of rapidly producing complex structures (similar to silicon chips or living cells) with atomic precision and a small number of defects. Living nature solves this problem by using linear polymer templates encoding large volumes of structural information into sequence of hydrogen bonded end groups which can be efficiently replicated and which can drive assembly of other molecular components into complex supramolecular structures. In this paper, we propose a nanofabrication method based on a class of photosensitive polymers inspired by these natural principles, which can operate in concert with UV photolithography used for fabrication of current microelectronic processors. We believe that such a method will enable a smooth transition from silicon toward molecular nanoelectronics and photonics. To demonstrate its feasibility, we performed a computational screening of candidate molecules that can selectively bind and therefore allow the deterministic assembly of molecular components. In the process, we unearthed trends and design principles applicable beyond the immediate scope of our proposed nanofabrication method, e.g., to biologically relevant DNA analogues and molecular recognition within hydrogen-bonded systems.

Molecular electronics promises multiple benefits over contemporary semiconductor technology. A single molecule with size of <1 nm can perform functions more complex than the smallest transistor crafted from silicon crystal by photolithography measuring tens of nanometers. Molecular switches,^1,2^ rectifiers,^3^ transistors,^4^ memories,^5,6^ and motors^7^ can be produced cheaply in large quantities by well-established methods of organic synthesis.^8^ Molecular memristors^9^ promise neuromorphic computing with synapses consisting of less than 100 atoms. Molecular quantum cellular automata promise to replace metallic wires by local interactions between neighboring charges or spins, allowing denser integration and reducing dissipated heat. Photonic circuits based on coupled molecular excitons promise to execute quantum algorithms at optical frequencies.^10,11^ Nevertheless, assembling any of these molecular components into complex machines such as computers remains an unsolved challenge.

Currently, state-of-the-art experiments in the field of molecular electronics are dominated by scanning probe microscopy (SPM) related methods, which provide an invaluable tool to measure and control the state of individual molecules.^12−16^ But despite admirable advances in automatic atomic force microscopy (AFM) manipulation,^17^ SPM methods seem to be too laborious and too slow to ever produce complex molecular machines at industrial scale. The fundamental bottleneck of SPM-based nanofabrication is the necessity to write structural information one-molecule-at-a-time by a heavy and slow macroscopic cantilever.

Therefore, self-assembly driven by noncovalent interactions currently represents the only viable method for building organized molecular structures at large scale. Self-assembling^18,19^ or crystal engineering^20^ can efficiently produce large scale regular structures (e.g., lattices,^21^ fractals^22^) by annealing the system toward the thermodynamic minimum. However, the variety and complexity into which small molecules can self-assemble are limited by the low amount of structural information that can be encoded into interactions between a few functional groups on the surface of these molecules. In addition, the selection of these functional groups can interfere with the operation of such molecular switches. From a design perspective, it is therefore desirable to decouple functional components from structural components in such molecular circuits.

In living nature, this problem is elegantly solved by introducing dedicated structural polymer scaffolds and templates (such as DNA, RNA, and proteins) which encode large amounts of structural information into long sequences of hydrogen bonding groups. Such polymers can therefore self-assemble into complex shapes as well as drive self-assembly or templated synthesis of other smaller molecules.^23,24^ These biochemical principles were exploited in artificial DNA-origami,^25−28^ which is currently the only technique capable of mass producing large quantities of molecular nanostructures with predetermined complex nonperiodic shapes.

In this paper we suggest combining bottom-up nanofabrication principles used by living nature with top-down photolithography used by the semiconductor chip industry. Namely, we aim to combine self-assembling driven by hydrogen-bonded polymer templates to organize the fine-structure of molecular circuits (at scale <10 nm) with photolithography methods capable of laying out arbitrarily complex structures of contemporary chips at mesoscopic scale (>10 nm). For this purpose, we are designing photosensitive polymer templates constituting complementary hydrogen-bonding functional groups similar to nucleobases attached to a diacetylene backbone.

The proposed nanofabrication method builds upon advances in DNA origami^28^ and exploits the discovery of diacetylene derivatives which can be efficiently polymerized in ultrahigh vacuum on insulating substrates^29^ when stimulated by UV light or by injection of electrons using a scanning tunneling microscopy tip.^30^ Such a general nanofabrication method can continuously scale from contemporary SPM-based molecular electronics experiments up to industrial mass production of circuits integrated with contemporary chip-manufacturing technology.

Our work focuses predominantly on assembling molecular components on ionic substrates, as such substrates are currently considered most suitable for unperturbed operation of molecular electronics and photonics.^11,31,32^ In particular, we envision the possibility of self-assembling complex quantum molecular cellular automata^33^ using either local reorganization of charges or coupled Frankel excitons to perform logical operations.^10,11^

In order to find an optimal photosensitive polymer assembler able to operate in this environment, we conducted a broad screening of possible molecular designs of complementary hydrogen bonded end groups that drive the self-assembly. In the future, we plan to optimize the interaction of the polymer with the ionic substrate and use various ionic substrates as templates for aiding the self-assembling.

## Photoassembling Nanofabrication Process

A major challenge of any bottom-up nanofabrication procedure is to meet two opposite requirements: (i) On the one hand, we desire high stability of the final supramolecular structure, which means that components must be strongly bonded, ideally by covalent bonds. (ii) At the same time, we need to produce these structures with a minimum number of defects at low temperatures since higher temperatures may damage the delicate chemical structure of functional molecular components. Therefore, the assembling process must be reversible at low temperature to allow low-temperature annealing, which means low binding energies. Difficulty meeting these two requirements makes production of defectless covalent organic frameworks so challenging.^18,34,35^

The essential trick used in nature to meet both of these contradictory requirements is to assemble the components using highly selective but weak supramolecular interactions and only then permanently join them by chemical reactions forming covalent bonds. High selectivity of the supramolecular interactions is essential not only to temporarily bind proper components together but also to preorient them in an optimal configuration for the subsequent covalent reaction. This is the essence of enzymatic catalysis, but the same principle was exploited also in artificial template-assisted synthesis.^24,36,37^ In nature, however, the covalent reaction is typically still thermally activated.

Here we propose to use electrocyclic reactions activated by nonthermal drivers to further enhance the potential of templated synthesis to form complex large-scale structures with small number of defects at low temperature. To make the reaction easily controllable by light and to make the nanofabrication process compatible with other chip-manufacturing processes, we design molecular assemblers which, unlike DNA, operate on the surface of a solid crystal in an anhydrous environment.

Derivatives of diacetylene molecules which were demonstrated to polymerize by UV light or electron injection on ionic substrates in a vacuum environment^29,30^ are the essential ingredient to make this possible. Photosensitivity of these reactions allows the use of state-of-the-art UV photolithography to imprint large scale structures of a circuit (>10 nm), while atomically precise placement of molecular components (<10 nm) is ensured by noncovalent self-assembling. The lattice constant of the polydiacetylene backbone (5.0 Å) matches almost exactly the distance between the 1,8-substituent positions in anthracene (4.94 Å, i.e., two lattice constants of graphene along the zigzag edge), as well as the distance between two β-substitution positions in porphyrin (5.1 Å). Therefore, polydiacetylene based templates can form large-scale commensurate self-assembled structures with many functional molecules considered as components for molecular electronics.

**Figure 1:**
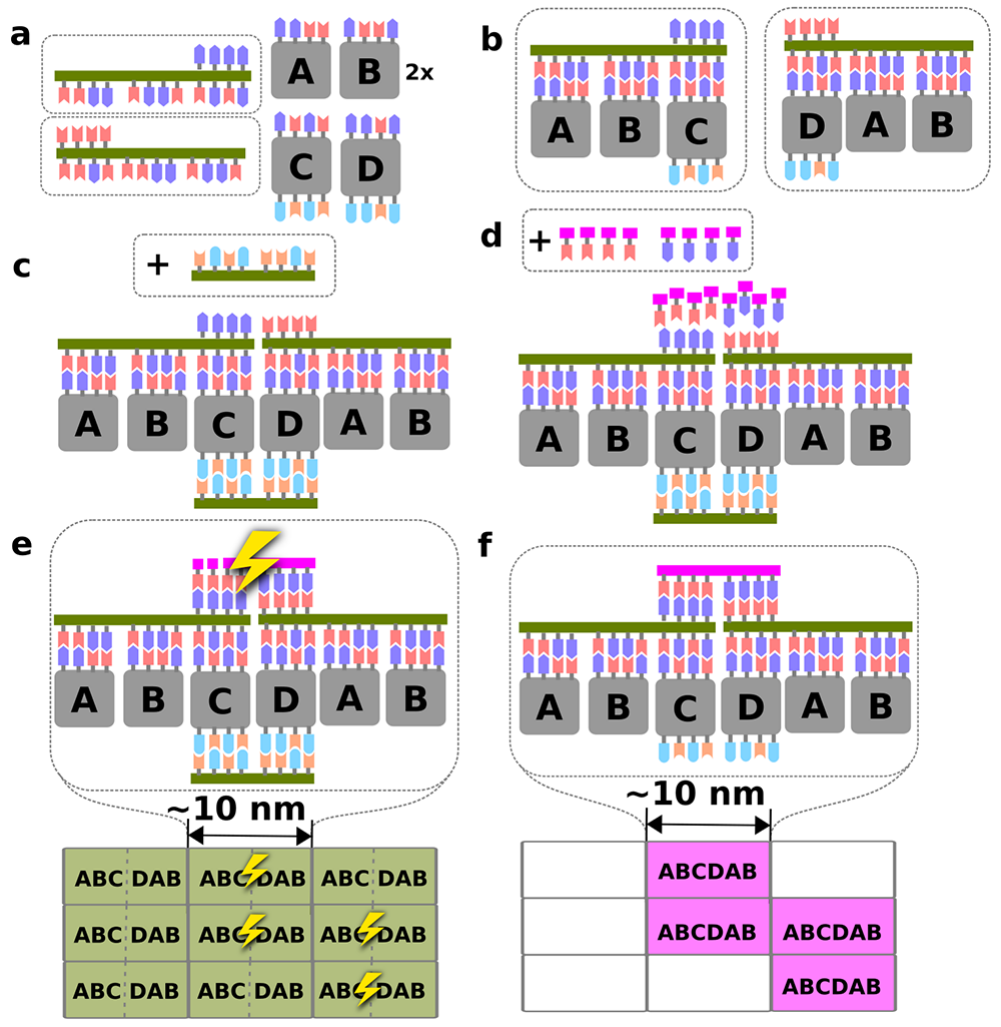
Schematics of the photoassembling process. (a) Functional molecular components (A, B, C, and D) decorated by complementary hydrogen bonding end groups (X in blue, Y in red) and oligomer templates (which encode the structure) are deposited on the substrate. (b) Components A, B, C, and D self-assemble with the templates forming ABC and DAB fragments. (c) Another oligomer template with weakly bonding end groups (X′ in cyan, Y′ in orange) is used to selectively join the two fragments into the ABCDAB structure. (d) Free monomers of X and Y self-assemble into rows along the remaining unpaired groups on templates. (e) The monomers are polymerized by UV light under a photolithography mask. (f) The temporary binder oligomer dissociates and is washed away. After washing, only the polymerized areas irradiated by UV light remain.

**Figure 2:**
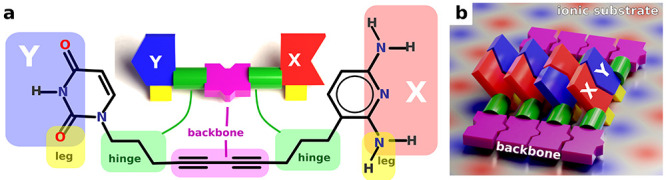
Illustration of a DNA analogue capable of photopolymerization. (a) Example of the chemical structure of a monomer with schematics of the functional modules. (b) 3D cartoon of a short polymer fragment which presents π–π stacking end groups (X,Y) oriented perpendicular to the substrate, binding to the ions by “legs”.

The proposed nanofabrication process consists of the following steps (depicted in Figure 1):

1. A mixture of several molecular electronics components and oligomeric molecular templates (i.e., short sequences of photo assembler) is deposited on the ionic substrate (panel a).
2. The system is annealed at a mild temperature forming a heterogeneous but regular self-assembled layer where there is selective supramolecular interactions between oligomeric molecular templates and the molecular electronics components (panel b). In this layer, the components are reversibly coupled by weak but highly selective noncovalent interactions with temporary binders fixing them in a specific place producing an atomically precise pattern on the ionic substrate (panel c). The units of the pattern (ABCDAB in Figure 1) are arranged into a regular lattice on a substrate by weak intermolecular interactions (as depicted in panel e). The complexity of such a pattern is limited by the information capacity of the templates, as well as by the speed of diffusion and annealing processes, which is significantly slower for large molecules. Therefore, we expect that up to a dozen of different molecular components can be simultaneously assembled in one step forming self-assembled patterns tens of nanometers in size.
3. Free photopolarizable monomers are added to the system (panel d) before the layer is irradiated by UV light using a photolithography mask. The irradiation then induces polymerization of the monomers into a permanent binder only in the areas exposed to the light (see the lighting symbol in panel e). This process produces a thermally stable and covalently interlinked framework. The coupling between the end groups in both permanent binders (red and blue) and temporary binders (cyan and orange) is purely noncovalent; therefore the assembling process should not affect the chemistry of the functional components. However, the end groups with stronger hydrogen bonds are chosen for the permanent binder. In the following, we discuss in detail the selection and tuning of binding energy of these end groups. The photoassembled end groups are more stable compared to the groups in the temporary binder, which allows UV light to transform (temporary) structures produced by reversible assembling into significantly more stable (permanent) structures.
4. The remaining nonpolymerized units (ABCDAB in a green box) which were not exposed to UV light are washed away by solvent or thermally evaporated at low pressure (panel f). This is possible due to the reversibility of weak noncovalent bonds between the components and the temporary binder. In contrast, the photopolymerized units (ABCDAB in a pink box) cannot be dissociated by a slight increase of temperature because the noncovalent bond formed in permanent binders (red and blue pairs) has a higher binding energy than the noncovalent one present in the temporary binder (cyan and orange pairs). In addition, the photopolymerized units are bigger and have a lower configurational entropy than the nonpolymerized ones, making them hard to wash away by solvent or to evaporate at low pressure. The monomers, photopolymerized into a permanent binder, can also be modified (e.g., by “legs” depicted in yellow in Figure 2) to enhance the anchoring of the photopolymerized units to the substrate. Nevertheless, the detailed discussion of tuning the interactions with the substrate is beyond the scope of this article.
5. In a way similar to the chip manufacturing process, this procedure can be repeated in multiple cycles in order to build more complicated multilayer structures composed of a large number of different molecular components.

## Design Criteria and General Layout of the Self-Assembler

The goal is to find self-assemblers with at least two complementary functional end groups (X,Y) attached to a common backbone, which (i) assembles deterministically and reliably into complementary pairs (X–Y only, never X–X or Y–Y) on an ionic substrate in vacuum under mild experimental temperatures (e.g., around room temperature) and (ii) can be efficiently polymerized under these conditions by UV light without damaging or altering the self-assembled structure. For the backbone, we consider diacetylene to be an ideal candidate as it was shown to spontaneously self-assemble into long rows driven by π–π stacking and polymerize by UV light even at cryogenic temperatures without structural changes (namely, the lattice constants of the stacked monomers and of the polymer are very similar, ∼5 Å).

Therefore, the main design variable is the choice of the end groups which should form well-defined complementary pairs with suitable binding energy, with minimal propensity of forming alternative binding motifs similar to noncanonical DNA structures.^38^ In essence, this means to avoid end groups prone to tautomerize or to form catemeres.^39^ For our initial design, we learned from nature and stuck to planar aromatic heterocycles analogous to DNA nucleobases. The combination of aromatic end groups with the diacetylene backbone (with lattice constant of ∼5 Å) automatically enforces the π-stacking of the end groups perpendicular to the substrate (and to the backbone), similarly as was observed for benzoic-acid groups attached to diacetylene polymerized on calcium carbonate.^29^ Designing the end groups such that they stand on the substrate (as opposed to laying on it) allows better packing of the polymer, therefore increasing the density of groups in a surface unit. Such a constrained general layout significantly simplifies the design and limits the possibility of unexpected binding modes.

## Results and Discussion

### Selection of Candidate End Groups

We generate a variety of aromatic heterocyclic end groups analogous to nucleic acids capable of forming two or three hydrogen bonds between nitrogen and oxygen containing terminations. In the future, other atoms such as boron, fluorine, and other halogens may be considered. We limit the design to a maximum of three hydrogen bonds not only in order to stick to the DNA prototype but also because four bonds would result in excessive binding energies preventing the reversibility of the assembling, and at least two bonds are necessary to constrain the orientation of the end groups. Nevertheless, we also included end groups with just one single hydrogen bond to better see the trends and effects of the individual building blocks.

We consider hydrogen donors such as primary and secondary amine (−NH_2_, −NH−), and acceptors such as pyridinic group (=N−), and keto group (=O) incorporated into a conjugated π-system of hexagonal and pentagonal carbon rings. We intentionally excluded other groups such as −OH, =NH as they can behave ambiguously (both as donor and as acceptor of the hydrogen bond) which would lead to an unpredictable assembly. Even this limited design-space leads to hundreds of possible end group structures (as we tested by a simple generative algorithm). For simplicity, in this initial work we selected only 64 of those end groups which are more closely related to nucleobases, and which did actually demonstrate some systematic trends useful for a fine-tuning of the binding energies.

Similar to nucleobases, we split our selection into short groups consisting of one single hexagonal ring like pyrimidine bases (cytosine, uracil, thymine) and long groups comprising hexagon and pentagon rings like purine bases (adenine, guanine). We assume that the length of the end group will provide additional selectivity due to steric constraints in closely packed self-assembled structures, as is the case in natural counterparts. Nevertheless, to make our design more robust, we want to select pairs of end groups that prefer complementary pairing X–Y (over homo pairs X–X, Y–Y) due to energetic reasons alone, even before considering steric effects. For this reason, our primary figure of merit is the binding energy contrast (*E*_C_ = *E*_XY_ – (*E*_XX_ + *E*_YY_)/2). In order to find good candidates for end groups optimized for reversible assembling at certain temperature (i.e., melting temperature in the context of DNA assembling), we split the pairs of end groups into certain ranges of total binding energy (*E*_XY_) corresponding to a target melting temperature, and within such energy windows select those with the highest contrast *E*_C_.

### Screening

Already our modest selection of end groups leads to a rather large variety of possible combinations (64^2^ = 4096). In order to find the most stable hydrogen-bonded structure, we generated all possible hydrogen bonded configurations, resulting in ∼15 000 trial structures, as described in the Methods section. All structures were optimized using a fast semiempirical method with corrections for hydrogen bonds and dispersion interactions DFTB3+D3H5.^44^ Then, for each pair, the optimized structure with the highest binding energy was selected as an estimation of the global energy minimum for further processing.

**Figure 3:**
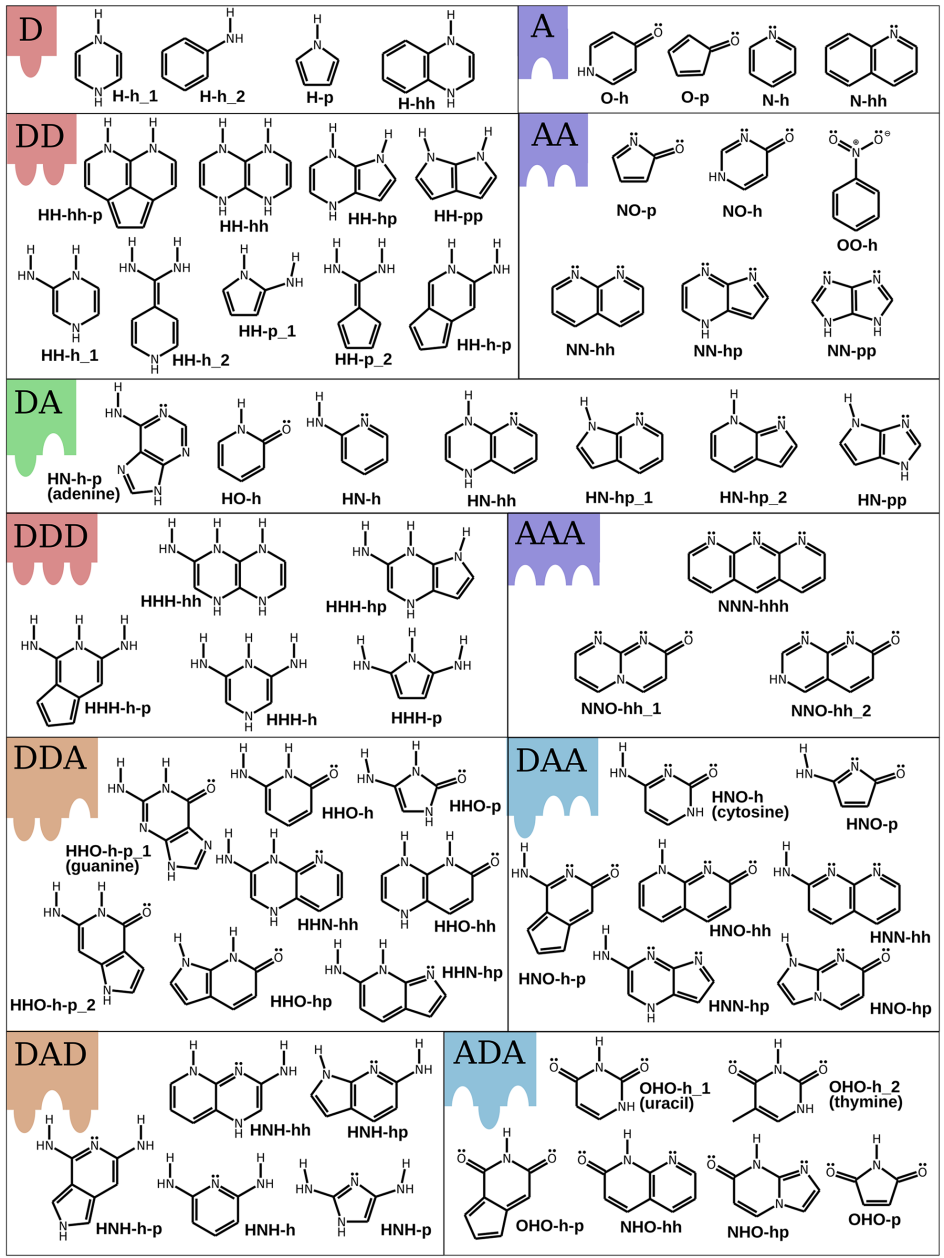
Assay of complementary hydrogen bonding end group candidates (nucleobase analogues) which we investigated in this article. Groups have 1–3 hydrogen bond donor (D) or acceptor (A) sites. We search for asymmetric groups forming preferentially heterogeneous pairs X···Y (e.g., D···A, DD···AA, DDD···AAA, DAD···ADA, DDA···DAA). Nevertheless, for completeness, we included the symmetric class DA. The assay contains all nucleobases (adenine, guanine, thymine, uracil, and cytosine) for reference. All molecules are named by shorthand codes with first capital letters denoting hydrogen donor (H) or acceptor sites (O, N) followed by hexagonal (h) or pentagonal (p) aromatic skeletons.

The results presented in Figure 3 show that both binding energy (blue, upper triangle) and contrast (red, lower triangle) show pronounced blocks of increased binding energy and contrast corresponding to complementary structure of hydrogen bond donor (D) and acceptor (A) sites. We call these structures “canonical pairs” (D···A, DA···AD, DD···AA, DDD···AAA, DDA···AAD, DAD···ADA) in resemblance to canonical (Watson–Crick) pairs in DNA. In the further analysis we paid special attention to these canonical base pairs. Additionally, the presence of noncanonical blocks with favorable binding energy, such as in the cases of DD···AAA, DDD···AA can be noted. All optimized structures and energies are included in the Supporting Information which may serve other researchers for further design of nucleobase analogues and investigation of general trends in hydrogen bonded systems.

### Trends and Design Rules

For the minimum energy configuration of these canonical pairs, we conducted a further refined geometry optimization using more demanding hybrid density functional theory (B3LYP-D3^46,47^), as described in the Methods section.

In order to find optimal base pairs for a certain temperature range, we ordered all refined geometries of the X···Y pairs by their binding energy and compared them to the binding contrast (see Figure 5). Since our goal is to find selective pairs with high contrast, we selected only pairs with contrast *E*_C_ > 5 kcal/mol for further analysis. By plotting binding energy, contrast, and various geometric parameters for all such pairs (see Supporting Information Figure S1), we were able to identify the most promising candidates and also to identify design-rules relating contrast EC to the geometric structure of the molecules. Figure 5 presents a summary of this analysis. In the figure, we report the 16 most promising candidates which provide maximum contrast in each range of energies and selected counterexamples of bad pairs. As one can see in Figure 5, the strongest bonds can be found for the class (DDA···AAD, green) analogous to the guanine-cytosine pair, which can be explained by multiple favorable cooperative interactions as was previously shown.^40^ Nevertheless, these “mixed” pairs (e.g., DDA···AAD, DAD···ADA) generally provide rather low contrast *E*_*C*_ due to their ability to form stable hydrogen bonded homo pairs. Therefore, it seems more promising to consider “pure” pairs (e.g., DDD···AAA, DDD···AA, DD···AAA, DD···AA) for rational design of nucleobase analogues, if we want to avoid formation of homo pairs and other noncanonical binding motives. In Figure 5 we selected 16 of the most selective complementary pairs that span an energy range between 7 and 23 kcal/mol with gaps no larger than 3 kcal/mol. This means that for our assembler design we can easily find a pair with binding energy matching the entropic term anywhere in this range, to target a specific melting temperature.

**Figure 4:**
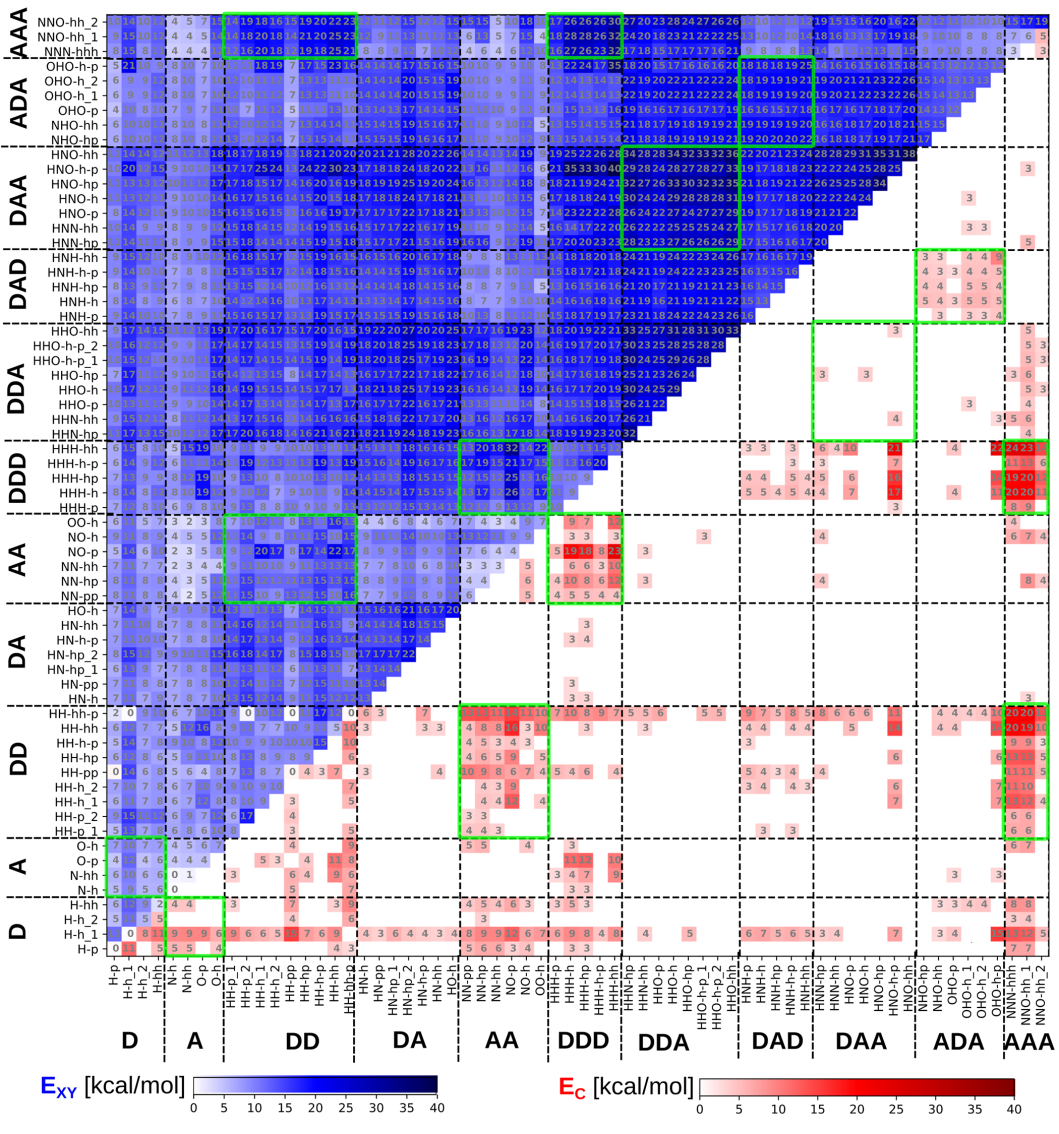
Binding energy and contrast were calculated with the DFTB3+D3H5 method. The blue upper triangle shows the total binding energy. The red lower triangle shows the energy contrast (only the base pairs with positive contrast are plotted). The gray number for each pair (box) is the value of the binding energy (contrast).

**Figure 5:**
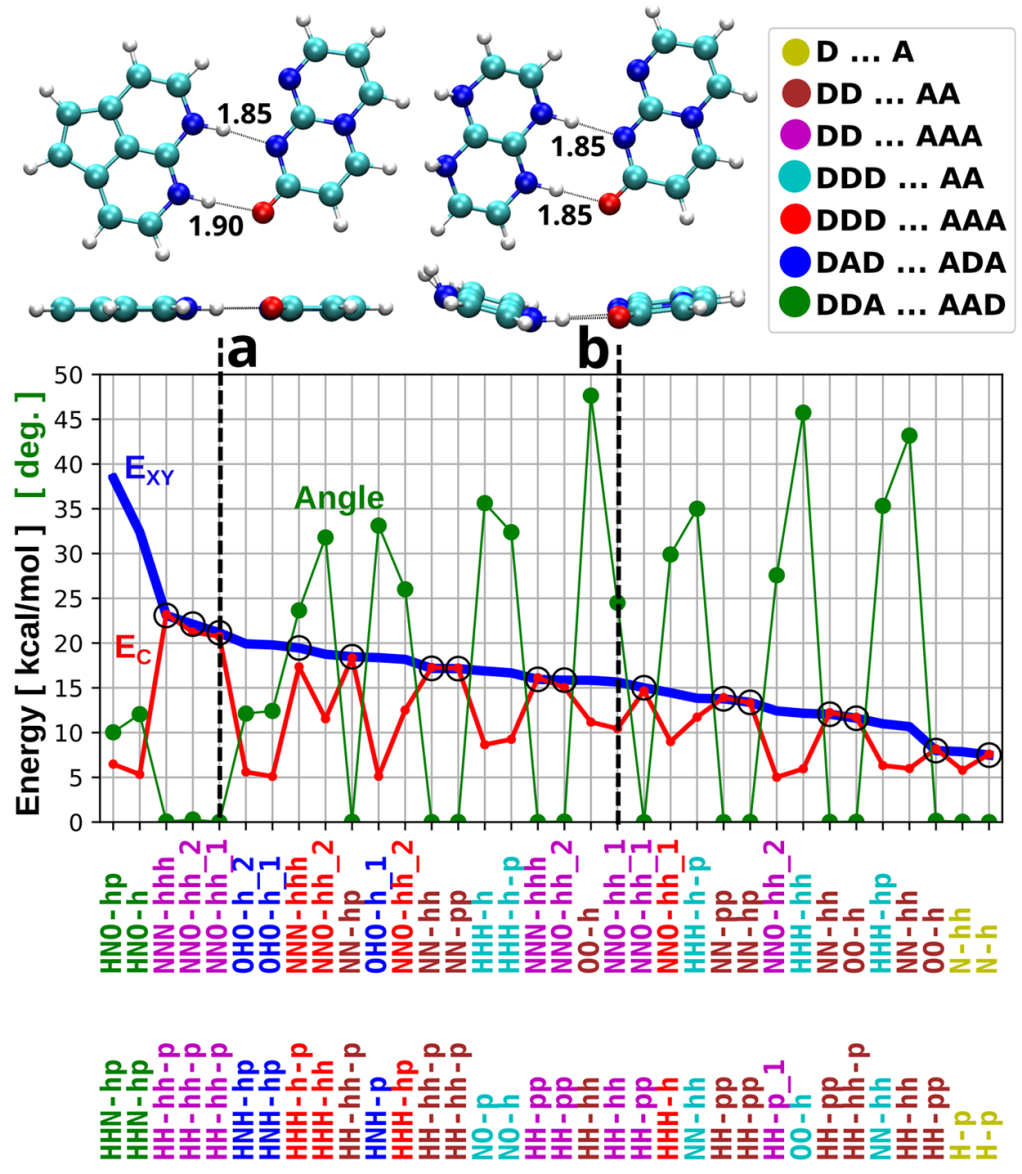
Binding energy (EXY, in blue) and contrast (EC, in red) for canonical pairs refined with the B3LYP-D3 method correlated with the dihedral angle of amino groups (in green). Out of 364 calculated configurations, only representative pairs for each group with contrast EC > 5 kcal/mol are shown. A more complete version of the figure is reported in the Supporting Information. The pairs with the planar configuration and highest possible contrast at different values of the absolute binding energy are marked with a circle. The correlation between contrast EC and dihedral angle is illustrated with two examples of similar pairs with (a) planar geometry and (b) a large dihedral angle.

Pure acceptors (A, AA, AAA) completely lack polarized hydrogens and therefore cannot form stable hydrogen bonded homopairs; moreover, the electronegative atoms with free electron pairs (−N=, =O) repel each other; therefore, the binding energy of homo pairs is close to zero. The situation is slightly more complicated for pure hydrogen bond donors (D, DD, DDD) which in our assay comprise primary and secondary amines (−NH_2_, −NH−). Amino groups are potentially capable of forming hydrogen bonds (similar to water) when a free electron pair of one group becomes an acceptor of hydrogen from another group. This is possible only when (i) the free electron pair is localized on the nitrogen and (ii) when it is geometrically accessible to the hydrogen donor. Both of these requirements are promoted by a pyramidal geometry of the amino groups and disrupted when it is conjugated to an aromatic system, which makes the amino group more planar with the electron pair delocalized into the aromatic system. In such planar configuration, the free electron pair is not accessible to hydrogen donors of neighboring molecules (assuming molecules are more-or-less fixed in the plane of the π-stacked structure).

**Figure 6:**
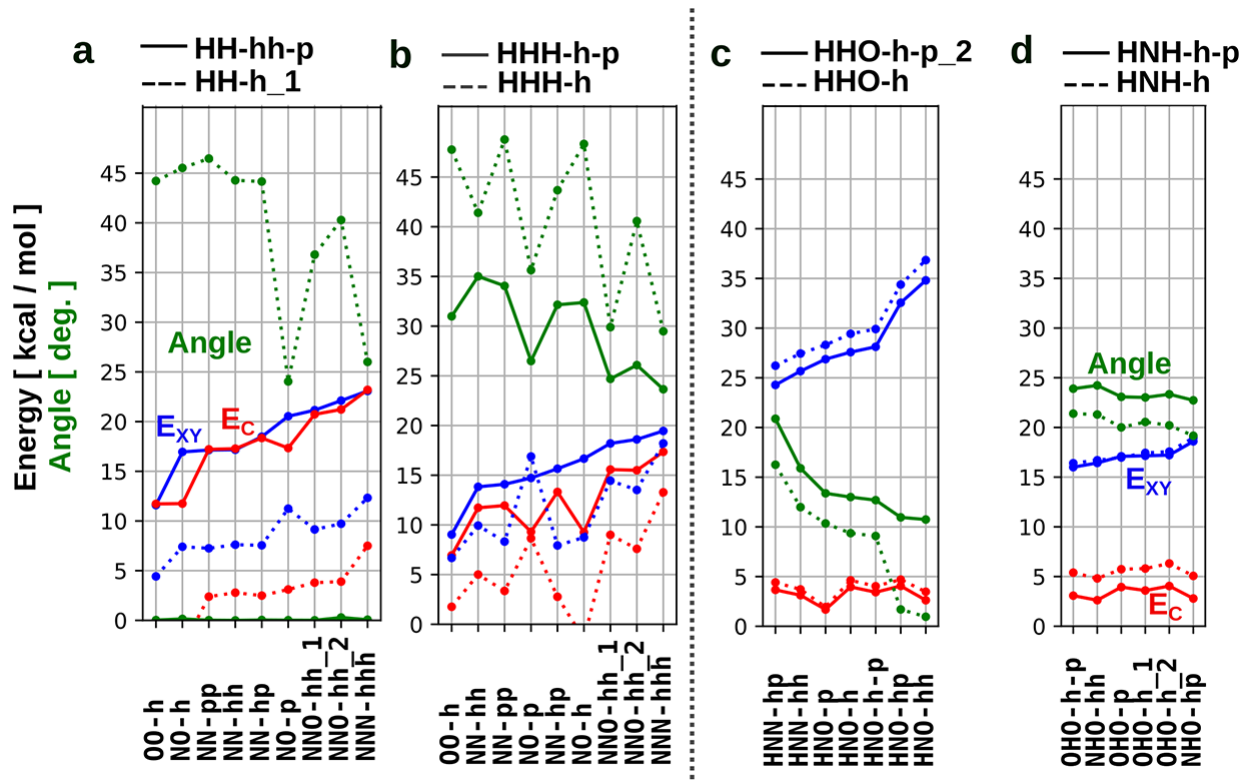
Trends and design rules. All figures show the correlation between the total binding energy of hetero pairs (EXY, blue), the binding energy contrast between hetero and homo pairs (EC, red), and the dihedral angle on amino groups (green). The trends are shown for selected representatives of the two “pure” classes (a) DD and (b) DDD, and “mixed” classes (c) DDA and (d) DAD.

Therefore, we found a strong correlation between the binding energy contrast *E*_C_ and the dihedral angle of the amino groups (see Figure 6, green vs red line). In essence, completely planar (i.e., highly conjugated) amino groups coincide with near zero binding energy of homo pairs between hydrogen bond donors. In fact, for these planar systems, the contrast *E*_C_ approaches the total binding energy of the hetero pair (*E*_XY_), since the binding energies of both donor and acceptor homo pairs are near to zero. For this reason, we consider the presence of a pure donor end group to be a decisive factor in designing reliable selective complementary pairs for nucleobase analogues. In our screening, the pure donor end groups HH-hh-p and HH-pp were found to be particularly good (selective, planar, strongly binding) donors (as shown in Figure 5). To further analyze these relations between binding energy and structure, we plot binding energy, contrast, and dihedral angle for selected base pairs in Figure 6.

In panel a, we report the profiles for all base pairs involving one of the most recurring end groups identified in Figure 5 (namely, HH-hh-p, together with another end group from the same class for comparison). The profiles for base pairs involving the HH-pp end group are provided in Supporting Information Figure S2. This is a pure donor; the hydrogen binding sites are planar, and the energy contrast profiles often approach the binding energy curves. Such molecules represent our primary choice of hydrogen donor end groups for the selection of optimal base pairs. In panel b, we report another candidate taken from the selection made in Figure 5. In this case, the dihedral angle on the amino groups is large, and this reflects on suboptimal values of the energy contrasts. For completeness, in panels c and d, the profiles for selected mixed pairs are plotted, where an inverse correlation between the binding energy and the dihedral angle can also be appreciated. In such cases, even if binding energies are quite large in absolute values, the selectivity of the interaction is rather limited, due to the above-mentioned possibility of forming strongly binding homo pairs. It is interesting to note that, for pure pairs, the size of the aromatic π-system strongly correlates with the planarity of the hydrogen binding sites. This is clearly visible by comparing short groups (pyrimidine-like, denoted by *-h) and long groups (purine-like, denoted by *-h-p). One can clearly observe that larger aromatic systems decrease the amplitude of the dihedral angle of amino groups (i.e., it makes the binding geometry more planar), which in turn correlates with a decrease of the binding energy and the energy contrast. This can be understood by a higher degree of conjugation and delocalization of the electron pair on amino groups into the larger π-system. Nevertheless, this trend is not visible in mixed pairs, which present similar binding energies irrespective of the size of the π-system. This means that selectivity based on steric considerations (i.e., complementarity of long and short nucleobases) can be seen as an independent (orthogonal) mechanism to achieve high selectivity, which does not interfere with the binding energy.

Planarity of the amino groups is also enhanced by the electron-deficient π-system, which “feed on” the free electron pair, and conversely amino groups are more pyramidal when other electron donating groups (including other amines) are conjugated to the π-system. This is the reason why groups such as HH-hh, HHH-h, HHH-hh turn out to be poor hydrogen-bond donors with low binding energy, low contrast, and high nitrogen dihedral angles. Based on these findings, we plan to further investigate ways to enhance conjugation of amino groups into the π-system, e.g., by engineering the size and aromaticity of the π-system and using electronegative substituents. We plan also to search for alternative donors which (unlike amino groups) lack the ability to behave as an acceptor at the same time (e.g., due to the lack of free electron pairs). This should help us in future computational designs of complementary nucleobase analogues, with strong preference of X–Y pairs and low propensity of forming alternative binding motives such as homo pairs or other unwanted bonding motifs.

### Implications for Design of DNA Analogues

Based on our exploration of 64 prospective end groups, we can conclude that even this small set densely samples the range of binding energy between 7 and 23 kcal/mol with gaps smaller than 3 kcal/mol with highly selective base pairs. This means that we can find an appropriate pair of highly selective complementary end groups to match any melting temperature corresponding to a specific entropic contribution in this energy range.

Nevertheless, for application purposes it would be interesting to combine several base pairs into one system (e.g., forming a 4 letter alphabet similar to A, C, T, and G in DNA). To achieve this, all letters in the alphabet have to prefer binding to a given partner sufficiently stronger than to all other alternatives (e.g., in DNA, guanine prefers to bind to cytosine over both homo pairs G···G and C···C, as well as hetero pairs G···A, G···T, etc.). To generalize the concept of binding energy contrast to nonbinary alphabets, we define it as the difference between the binding energy of a specific pair (*E*_XY_) and the maximum binding energy over all other alternatives, *E*_C_ = *E*_XY_ – max_*Z*_{*E*_XZ_, *E*_YZ_}. In order to check this, we needed to calculate the full interaction matrix (Figure 4) between all possible pairs.

**Figure 7:**
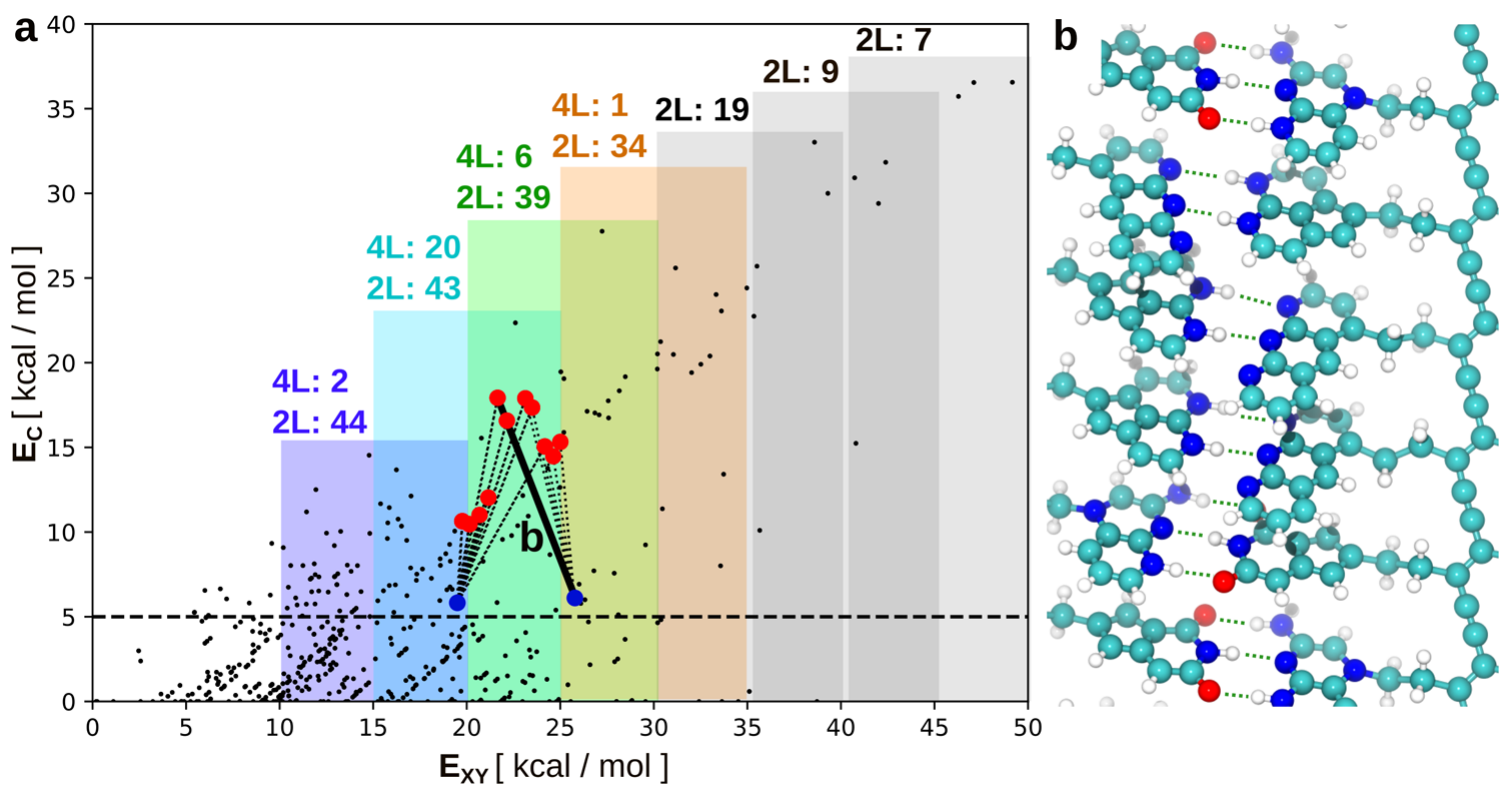
Summary of DNA analogue design opportunities. (a) Count of 2 letter (2L) and 4 letter (4L) alphabets with EC > 5 kcal/mol found in different binding energy windows evaluated from the binding matrix (Figure 4) based on DFTB+D3H5 calculations. Each dot represents an end group pair. The energy windows (shown as transparent blocks) move in increments of 5 kcal/mol and have a width of 10 kcal/mol. Energy windows containing at least one 4L alphabet are colored (from blue to red). Bold colored dots represent the pairs involved in the 4L letter alphabet. Each of the 4L alphabets (shown as thin dotted lines) is a combination of one pure (red) pair and one mixed (blue) pair. (b) Example of a polymerized and noncovalently paired sequence formed from one of the 4L alphabets (namely, HH-hh-p, NNN-hhh, HNH-hh, and OHO-h-p). The corresponding pair is highlighted with the bold line and letter b in panel a.

Currently we have these data only at the DFTB+D3H5 level of theory. Therefore, our results in this respect are only preliminary. Nevertheless, we can still make qualitative conclusions about the distribution of such nonbinary alphabets. In Figure 7a we present the contrast *E*_C_ plotted with respect to the binding energy *E*_XY_ for all respective pairs as calculated using the DFTB+D3H5 method. We count all possible alphabets that provide *E*_C_ > 5 kcal/mol and exclude end groups with just one hydrogen bonding site. As one can see, a larger number of binary alphabets (2L) can be found for lower binding energies (e.g., 44 alphabets for binding energy between 10 and 20 kcal/mol vs only 34 alphabets in the 25–35 kcal/mol window). This can be understood simply by the lower density of pairs with a larger binding energy, as visible from the plot in Figure 7a. In contrast, only two 4L alphabets can be found in the region 10–20 kcal/mol vs 20 4L alphabets in the region 15–25 kcal/mol. In fact, all such alphabets appear in a narrow range of binding energies around the 20–25 kcal/mol interval, as can be seen in Figure 7a.

It is interesting to notice that all 4L alphabets found are made of one pure (e.g., DDD···AAA, DD···AA, etc.) and one mixed (e.g., DDA···AAD, DAD···ADA) base pair. This is not surprising considering that any pure donor binds rather strongly to any pure acceptor, which hinders the formation of an alphabet with two selective (i.e., mutually exclusive) pairs made of only pure end groups. Among the mixed groups, the pairs with DAD···ADA structure exhibit higher selectivity than DDA···AAD. This could be because DDA or AAD end groups can bind more strongly to pure end groups due to better geometric accessibility, which decreases the binding contrast. In fact, all 4L alphabets found rely on a small subset of end groups, namely HNH-hh in the DAD group; OHO-h-p, OHO-h_1 and OHO-h_2 in the ADA group; HH-hh-p, HH-hh, HH-hp, HH-h_1 and HH-h_2 in the DD group; NO-h-p and NO-p in the AA group; and NNN-hhh and NNO-hh_1 in the DDD group, as highlighted in Table S3 in the Supporting Information. In our opinion, future works should focus on this subset, possibly by including modifications of these molecules.

Sadly, we did not find any six-letter (6L) or larger alphabet for any 10 kcal/mol binding energy window from end groups present in our computational assay. This is again understandable considering that all 4L alphabets combine one pure pair and one mixed pair. Forming a larger alphabet would probably require introducing another distinct class of end groups that do not bind strongly to the groups already present in 4L alphabets. This can be eventually achieved in future either by employing a steric mechanism of selectivity (i.e., long vs short base pairs) or by introducing other types of noncovalent bonding such as halogen bonds providing an alternative (orthogonal) mechanism of selective binding.

Finally, to illustrate how the final structure of the polymers terminated with selective end groups looks like, in Figure 7b we plot the 3D rendering of a possible atomic arrangement of the 4L alphabet made of the HH-hh-p, NNN-hhh, HNH-hh and OHO-h-p molecules. In this representation, the end groups are chemically bonded to the diacetylene backbone via a dimethylene linker. In the panel, hydrogen bonds are depicted with green dashed lines, highlighting the presence of short (i.e., HNH-hh and NNN-hhh) and long (HH-hh-p and OHO-h-p) end groups.

## Conclusions

In this article, we have proposed a nanofabrication scheme which combines two major approaches (i.e., a photolithographic top-down process and a self-assembling bottom-up one) into a single tool: a photopolymerizable analogue of DNA origami. In order to prove the feasibility of the concept, we have explored 64 candidates for hydrogen bonding end groups analogous to DNA nucleobases which are capable of providing complementary binding (X···Y preferred over X···X and Y···Y) with deterministic geometry. Calculations using dispersion corrected hybrid density functional theory (B3LYP-D3) demonstrated that such pairs of end groups can be found for a rather broad range of binding energies (between 7 and 23 kcal/mol) which allows us to optimize the polymer template for different operating temperatures and entropies of the backbone. This potentially allows the design of hierarchical self-assembling protocols where stronger hydrogen-bonded pairs are preserved while weaker pairs anneal or dissociate (such as depicted in Figure 1). We have also found larger alphabets containing 4 rather than 2 complementary end groups, although this was done only on the level of empirically corrected density functional tight binding (DFTB+D3H5) which have to be validated by more accurate methods. On a more practical ground, we were able to identify 16 promising candidate pairs (and a few key end groups) which can be further considered for studying the interaction with the substrate (with ad hoc modifications to be introduced) and their stacking to form the final packed structure reported as an example in Figure 7b. Moreover, narrowing down the selection of end groups allows us to further the computational design of the photopolymerizable templates by studying the effect of different linkers (“hinge” in Figure 2) on binding entropy and melting temperature of the polymer and introduce polar functional groups which will control binding of the molecule to the ionic substrate (i.e., “leg” in Figure 2). In broader terms, our study establishes an effective framework for computationally screening highly selective nucleobase analogues in π-stacked hydrogen bonded systems, and it identifies guidelines for the *ex novo* design of such molecules. In particular, we found that the degree of conjugation and the planarity of amino groups in the hydrogen bonding sites play a critical role for forming well behaved hydrogen bonded base pairs with high selectivity. We believe that these principles can be employed beyond the immediate scope of our proposed nanofabrication method, e.g., in the context of biologically relevant DNA analogues and molecular recognition within hydrogen-bonded systems, in general.

## Methods

The set of candidate molecules was built using DNA nucleobases as templates and by adding modifications that would preserve the planarity of the end groups, resulting in 64 structures as reported in Figure 3. All molecules were sketched using the Avogadro software^41^ and preoptimized according to the UFF force field.^42^ After that, the structures were further optimized using the density functional based tight binding (DFTB) method, as implemented in the DFTB+ code.^43^ For all DFTB calculations, we employed the D3H5 corrections^44^ (which were developed in order to properly describe hydrogen bonded systems), with a force convergence criterion of 0.12 (kcal/mol)/Å. The optimized end groups were then used to find all possible pairs by placing them at a distance of 2 Å along the hydrogen bond direction. We considered all possible combinations of assembly by shifting and flipping the molecules along the hydrogen bond direction. This means, for example, that for two asymmetrical end groups with three hydrogen bond sites, we considered 10 initial configurations (i.e., 4 poses with one1ydrogen bond, 4 with two bonds and 2 with three bonds, see Figure S4 in the Supporting Information). In such a way, we obtained ∼15 000 configurations, which were then optimized using the DFTB method. For each pair of molecules, we selected the binding pose with the minimum energy and consistent with a planar geometry. The binding energy *E*_XY_ is calculated from the difference between the energy of the pair and the sum of the energies of isolated end groups. The energy contrast *E*_C_ is computed as the difference between the binding energy of the pair and half of the sum of the binding energies of the homo pairs. Selected pairs were further refined by means of the Density Functional Theory (DFT) method, as implemented in the PSI4 code,^45^ using the B3LYP-D3^46,47^ functional and the cc-pVDZ basis set. To minimize errors introduced due to the limited basis set, counterpoise-corrected basis-set superposition error (BSSE) corrections^48^ were considered. The binding energies discussed throughout this paper were calculated by subtracting the energies of the two independently optimized end groups from the BSSE-corrected energy of the complex (we call it “BE_relax” in the Supporting Information). For completeness, we also provide the BSSE-corrected interaction energy of the two rigid fragments (i.e., unrelaxed end groups, “BE_rigid” in the Supporting Information) as given by the PSI4 program because this quantity is also often reported in the literature. The method and basis set were chosen based on the benchmark published in the BioFragment Database,^49^ where B3LYP-D3/cc-pVDZ was found to be the cheapest method providing chemical accuracy (1 kcal/mol) for the hydrogen bonded subset of the S22 data set.^50^ We also considered the counterpoise-corrected basis-set superposition error corrections. The QCHEM and density-fitting (DF) criteria were used for geometry and SCF convergences, respectively. The pyramidal angle reported in Figure 6 is the average of improper dihedral angles centered at the nitrogen atoms in the amino groups involved in the hydrogen bonds.

## Supporting Information Available

Figure S1 showing binding energies, contrast, and dihedral angles for all B3LYP-D3-refined configurations with contrast larger than 5 kcal/mol; Figure S2 showing more complete version of Figure 6; Table S3 listing combinations of end groups that form a four-letter alphabet; Figure S4 showing example of trial configurations considered for the screening (PDF)
Data set containing all end group and base pair configurations optimized with both B3LYP-D3 and DFTB+D3H5 methods (ZIP)
