# Supplementary material for: Computational Design of Photosensitive Polymer Templates To Drive Molecular Nanofabrication
Source: ACS Nano. 2024 Mar 28;18(14):9969–79. doi: 10.1021/acsnano.3c10575 (PMC11008366; doi:10.1021/acsnano.3c10575)
Supplement: Supplementary file 1 — nn3c10575_si_001.pdf [file nn3c10575_si_001.pdf]

Supporting Information of

Computational design of photosensitive polymer templates to drive molecular nanofabrication

*Mithun Manikandan, Paolo Nicolini, Prokop Hapala**

Institute of Physics (FZU), Czech Academy of Sciences, Na Slovance 2, 182 00 Prague, Czech Republic

* hapala@fzu.cz

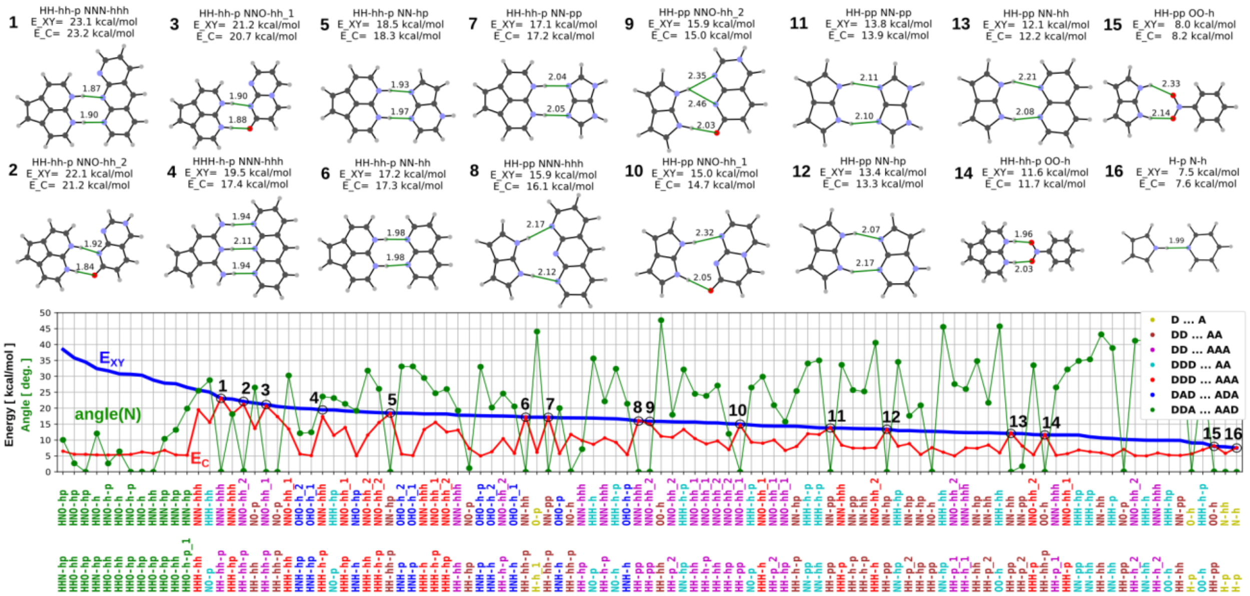

Figure S1. Binding energy and contrast for canonical pairs refined with the B3LYP+D3 method, correlated with the dihedral angle of amino groups. Out of 364 calculated configurations, only those with contrast E_C > 5 kcal/mol were selected. Out of these we selected 16 with the highest contrast in its binding energy range. The optimized geometry of these 16 selected base pairs is plotted above (with hydrogen bond-length in Å shown).

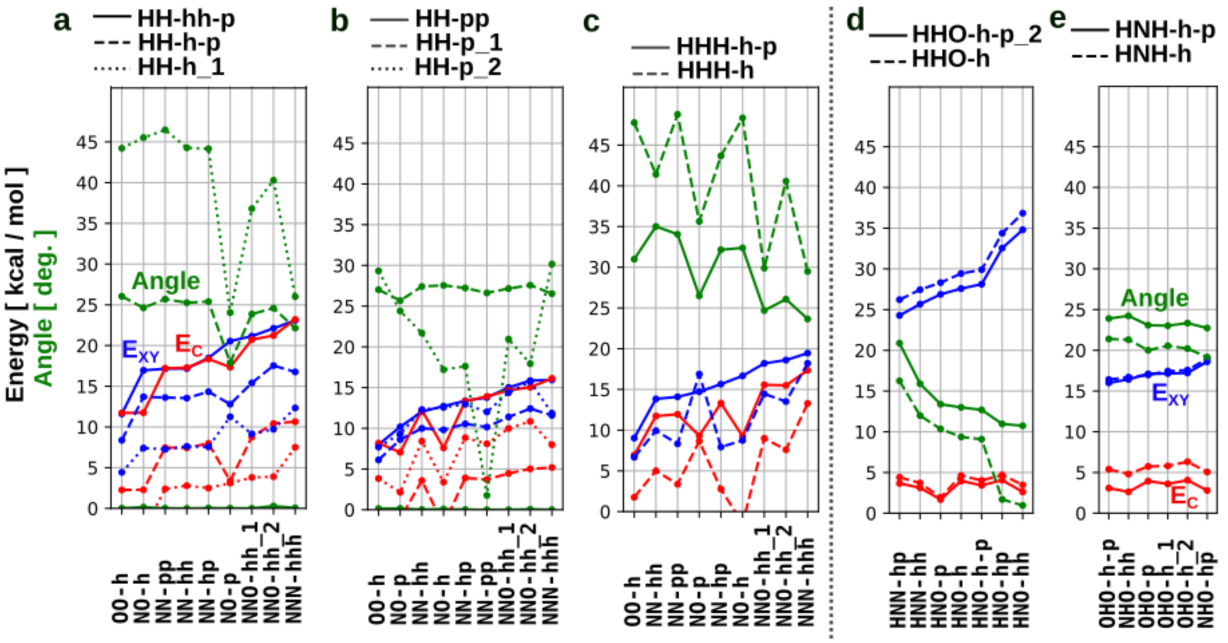

Figure S2. Correlation between total binding energy of hetero pairs (E_{XY}, blue), the binding energy

contrast between hetero and homo pairs (E_C, red) and the dihedral angle on amino groups (green). The trends are shown for selected representatives of the two "pure" classes a) and b) DD, c) DDD and "mixed" classes d) DDA and e) DAD.

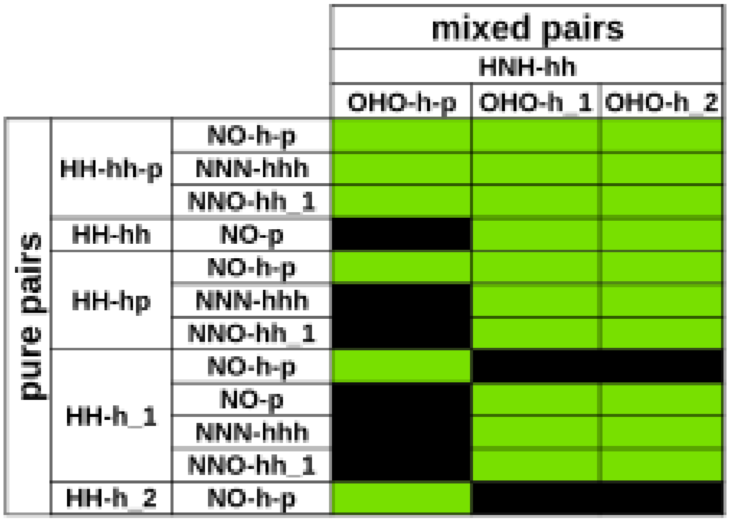

Table S3. All possible combinations of end groups which form a four-letter alphabet. Please refer to the main text for more details.

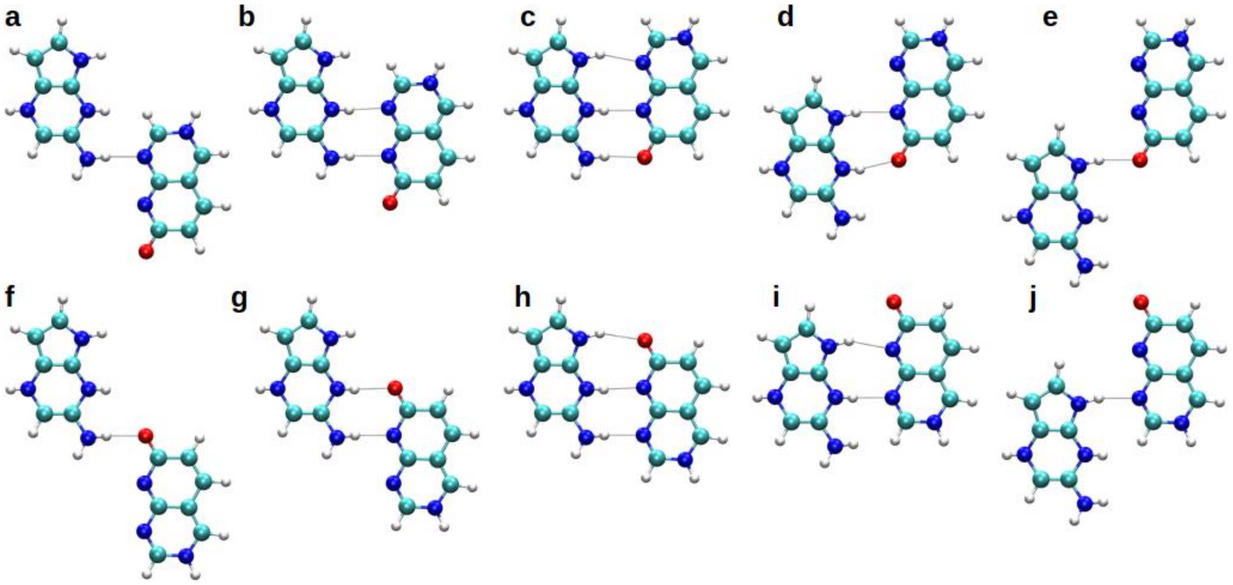

Figure S4. All possible combinations of assembling for the HHH-hp..NNO-hh end group pair. In this case the the two end groups are asymmetric, therefore one needs to consider not only the

configurations obtained by shifting one end group with respect to the other (panels a-e), but also those formed with one molecule flipped along the hydrogen-bond direction (panels f-j).
